# Local Anaesthesia

**Published:** 1877-06

**Authors:** 


### EDITORIAL.

### LOCAL ANÆSTHESIA.

The application for Local Anæsthesia invented by Dr. Von Bonhurst, has recently been much improved by the substitution of felt cones instead of the sponges for holding and applying the anæsthesia liquid.

They are more convenient, being ready for use when they come to hand and can be used with greater precision than the sponges.

